# Maternal Aerobic Exercise, but Not Blood Docosahexaenoic Acid and Eicosapentaenoic Acid Concentrations, during Pregnancy Influence Infant Body Composition

**DOI:** 10.3390/ijerph19148293

**Published:** 2022-07-07

**Authors:** Cody J. Strom, Samantha M. McDonald, Mary-Margaret Remchak, Kimberly A. Kew, Blake R. Rushing, Joseph A. Houmard, David A. Tulis, Roman Pawlak, George A. Kelley, Lisa Chasan-Taber, Edward Newton, Christy Isler, James DeVente, Madigan Raper, Linda E. May

**Affiliations:** 1 Department of Kinesiology and Sport, University of Southern Indiana, Evansville, IN 47712, USA; 2 School of Kinesiology and Recreation, Illinois State University, Normal, IL 61790, USA; smmcdo4@ilstu.edu; 3 Department of Kinesiology & Health, Rutgers University, New Brunswick, NJ 08901, USA; mer253@scarletmail.rutgers.edu; 4 Department of Biochemistry and Molecular Biology, Brody School of Medicine, East Carolina University, Greenville, NC 27834, USA; kakaplan@fbi.gov; 5 Department of Nutrition, Nutrition Research Institute, University of North Carolina-Chapel Hill, Kannapolis, NC 28081, USA; blake_rushing@unc.edu; 6 Department of Kinesiology, College of Health and Human Performance, East Carolina University, Greenville, NC 27858, USA; houmardj@ecu.edu (J.A.H.); mayl@ecu.edu (L.E.M.); 7 East Carolina Diabetes and Obesity Institute, East Carolina University, Greenville, NC 27858, USA; 8 Department of Physiology, Brody School of Medicine, East Carolina University, Greenville, NC 27834, USA; tulisd@ecu.edu; 9 Department of Nutrition Science, East Carolina University, Greenville, NC 27858, USA; pawlakr@ecu.edu; 10 Department of Epidemiology and Biostatistics, West Virginia University, Morgantown, WV 26505, USA; gkelley@hsc.wvu.edu; 11 Department of Biostatistics & Epidemiology, University of Massachusetts, Amherst, MA 01003, USA; lct@schoolph.umass.edu; 12 College of Obstetrics and Gynecology, Brody School of Medicine, East Carolina University, Greenville, NC 27834, USA; newtoned53@gmail.com (E.N.); islerc@ecu.edu (C.I.); deventeja@ecu.edu (J.D.); 13 Edward Via College of Osteopathic Medicine, Blacksburg, VA 24060, USA; mraper@vt.vcom.edu

**Keywords:** pregnancy, exercise, DHA, EPA, infant, body composition

## Abstract

Although discrete maternal exercise and polyunsaturated fatty acid (PUFA) supplementation individually are beneficial for infant body composition, the effects of exercise and PUFA during pregnancy on infant body composition have not been studied. This study evaluated the body composition of infants born to women participating in a randomized control exercise intervention study. Participants were randomized to aerobic exercise (n = 25) or control (stretching and breathing) groups (n = 10). From 16 weeks of gestation until delivery, the groups met 3×/week. At 16 and 36 weeks of gestation, maternal blood was collected and analyzed for Docosahexaenoic Acid (DHA) and Eicosapentaenoic Acid (EPA). At 1 month postnatal, infant body composition was assessed via skinfolds (SFs) and circumferences. Data from 35 pregnant women and infants were analyzed via *t*-tests, correlations, and regression. In a per protocol analysis, infants born to aerobic exercisers exhibited lower SF thicknesses of triceps (*p* = 0.008), subscapular (*p* = 0.04), SF sum (*p* = 0.01), and body fat (BF) percentage (%) (*p* = 0.006) compared with controls. After controlling for 36-week DHA and EPA levels, exercise dose was determined to be a negative predictor for infant skinfolds of triceps (*p* = 0.001, r^2^ = 0.27), subscapular (*p* = 0.008, r^2^ = 0.19), SF sum (*p* = 0.001, r^2^ = 0.28), mid-upper arm circumference (*p* = 0.049, r^2^ = 0.11), and BF% (*p* = 0.001, r^2^ = 0.32). There were no significant findings for PUFAs and infant measures: during pregnancy, exercise dose, but not blood DHA or EPA levels, reduces infant adiposity.

## 1. Introduction

In 2018, 13.4% of 2–5-year-old children in the United States were obese [1]. Childhood obesity increases the risks of adult chronic health conditions, such as obesity, metabolic syndrome, diabetes, and heart disease [1]. Obese pregnant women tend to deliver offspring with a higher birthweight, subsequently increasing the risk of obesity across their lifespan [2,3]. Current evidence links gestational infants to the rising prevalence of obesity across the last 25 years [4,5,6,7], thus leading to a cycle of generational obesity [3]. 

Maternal aerobic exercise interventions protect against large infant birth weights among underweight, normal weight, overweight, and obese women [2,8]. Although some research focuses on infant birth weight, the focus may be better directed at regulating body composition (decreasing fat mass and increasing lean body mass). Clapp et al. showed that while infants born to women who engaged in moderate to high intensity aerobic exercise had increased birth weight, there was no change in fat mass, and an increase in lean body mass compared with the infants of controls [9,10]. Clapp et al. also demonstrated that the 5-year-old offspring of women who performed aerobic exercise during pregnancy had less fat mass, with similar muscle mass compared with infants born to the control group [11]. Current research has also shown that sedentary behaviors during pregnancy are associated with decreased fatty acid transport protein (FATP)2 and FATP3 mRNA, which are indirectly correlated with peroxisome proliferator-activated receptor gamma (PPAR-γ) in placental samples [12].

Maternal supplementation of Docosahexaenoic Acid (DHA) results in normal infant birth weight, decreased fat mass, and increased lean mass of preterm infants [13]. Similarly, maternal DHA supplementation during pregnancy is associated with decreased infant fat mass and increased lean body mass [13]. DHA and Eicosapentaenoic Acid (EPA) activate PPAR-α, which can inhibit expression of pro-inflammatory markers [14]. However, the potential influence of both maternal aerobic exercise and maternal polyunsaturated fatty acids (PUFAs) concentrations on infant body composition remains unknown.

Investigating the effect of both exercise and maternal PUFA supplementation on infant body composition can provide valuable knowledge related to the risk of childhood obesity and may influence perinatal recommendations. However, to our knowledge, current research has not investigated the relationship of maternal exercise and PUFA concentrations on infant body composition within the same study. Thus, the purpose of the current study was to determine the influence of prenatal aerobic exercise and maternal concentrations of DHA and EPA on 1-month-old infant body composition measures. Specifically, this study assessed independent and potentially synergistic effects of prenatal aerobic exercise and maternal DHA and EPA concentrations on 1-month-old infant body composition. We hypothesized that maternal aerobic exercise and higher DHA and EPA concentrations result in improved infant body composition (e.g., decreased BF%) compared with infants born to non-exercising controls with lower DHA and EPA concentrations. 

## 2. Materials and Methods

As part of a larger, longitudinal randomized controlled trial (ECUIRB#12-002425) this secondary post-hoc analysis was approved in accordance with the declaration of Helsinki by the East Carolina University Institutional Review Board and Vidant Medical Center Review Board. 

### 2.1. Study Design

We conducted a secondary analysis using data from the ENHANCED by Mom study that consisted of a 24+ week, prospective, partially blinded, randomized, controlled exercise intervention trial conducted between 2015 and 2018 [15,16]. The current study focused on evaluating the effects of prenatal exercise and maternal DHA and EPA concentrations on infant body composition at 1 month postnatal age. At 1 month after delivery, infant body composition was assessed via skinfolds, circumferences, morphometrics, and body mass index (BMI). 

#### 2.1.1. Participant Recruitment and Randomization 

Women that were pregnant with a singleton pregnancy were recruited from local obstetric clinics, flyers posted in local businesses, social media, and word-of-mouth in the eastern North Carolina area. Pregnant women were eligible for the study if they met the following criteria: (1) were 13 to 16 weeks pregnant; (2) had a singleton pregnancy; (3) were between the ages of 18 and 40; (4) had a pre-pregnancy BMI between 18.5 and 39.99 kg/m^2^; (5) were cleared to participate in an exercise intervention by their obstetric provider; (6) did not exhibit any chronic disease; and (7) were abstaining from alcohol, tobacco, illicit drugs, or any other medications that potentially have negative impacts to fetal growth. After consenting to the study and upon receipt of the obstetric provider clearance letter, participants engaged in initial testing to establish a baseline. The initial testing consisted of a submaximal exercise treadmill test and a fasted blood draw. Following the initial baseline measures, participants were assigned to either the aerobic exercise or the stretching and breathing group via sealed, sequentially numbered envelopes derived from computer-generated randomization (Graph-Pad).

#### 2.1.2. Participant Retention

**Figure 1:**
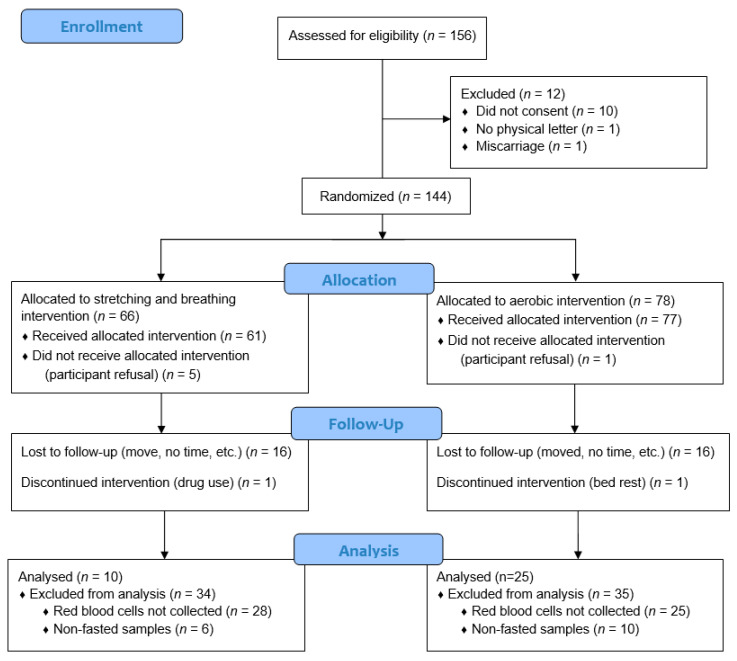
CONSORT Diagram.

A flow diagram of the participants through the trial is shown in Figure 1. Of the 156 pregnant women initially assessed for eligibility, 144 were randomized to the moderate-intensity aerobic exercise group (n = 78) or the control group (n = 66). Throughout the study, 40 participants were lost to follow-up as a result of the following: participant refusal (n = 6); moved, no time, or lost interest (n = 32); excluded due to drug use (n = 1); and discontinued due to bed rest (n = 1). Of the remaining 104 participants, 69 were excluded due to missing data for RBCs (n = 53) and non-fasted samples (n = 16), yielding a final sample of 35 pregnant women (aerobic = 25, control = 10) eligible for the intent-to-treat analyses. Six exercisers were not “exercise adherent.” Thus, the per protocol analysis at 1 month postnatal consisted of 19 aerobic exercisers and 10 control women.

### 2.2. Maternal Submaximal Treadmill Test

A submaximal treadmill exercise test was completed by all participants following the modified Balke treadmill (Trackmaster 425, CareFusion, Newton, KS, USA) protocol to 85% of heart rate (HR) maximum, previously validated in pregnant women [17]. Maternal measurements of carbon dioxide production and oxygen consumption were collected via indirect calorimetry (ParvoMedics, Salt Lake City, UT, USA) throughout the duration of the test; rating of perceived exertion (RPE) and HR were used to ensure the VO_2peak_ was achieved. From the HR and VO_2peak_ treadmill test results, estimated individual target heart rate (THR) ranges associated with moderate intensity exercise (40–59% VO_2peak_) and light-intensity (<30% VO_2peak_) for the control stretching and breathing group were calculated [17,18].

### 2.3. Exercise Intervention

Participants completed 3 days of exercise weekly from 16 weeks gestation until delivery based on their personal availability. Participants received one-on-one supervised exercise training for all exercise sessions. Trainings were supervised by trained/certified exercise physiologists who were study personnel specifically trained to work with pregnant women. Heart rate was tracked throughout each session using heart rate monitors (Polar FS2C HR monitor) to ensure participants were at the prescribed intensity. Heart rate (via Polar monitor) and blood pressure (via manual auscultation) were measured prior to and following each exercise session. All participants began their sessions with 5 min of light-intensity treadmill walking (speed ~3.0 mph), followed by 50 min of their prescribed protocol, then 2–3 min of cool-down. Neither the participant nor their exercise trainer could be blinded to group allocation due to the nature of the intervention.

#### 2.3.1. Aerobic Exercise Group

For the aerobic exercise group, each exercise session consisted of 50 min of continuous, moderate intensity aerobic exercise, using aerobic exercise equipment of the participants’ choice (e.g., treadmill, elliptical, rowing, cycle ergometer). Moderate intensity exercise was chosen in accordance with ACSM and ACOG guidelines [18,19,20]. A ramp up transition period of 2 weeks was assigned by starting participants at 30 min of moderate intensity exercise and then progressing 5 min in each exercise session until 50 min was achieved [21].

#### 2.3.2. Stretching and Breathing Comparison Group

For the stretching and breathing comparison group, each participant engaged in an exercise session that consisted of 50 min of guided stretching and breathing techniques. Stretches were prescribed to target all major muscle groups and breathing exercises were selected to focus on maintaining controlled inhalation and exhalation during each stretch. To ensure adherence to group randomization and to maintain light intensity (<30% VO_2peak_), participants in the stretching and breathing group also wore HR monitors which were frequently checked by the trained exercise physiologist. This type of attention control group helps to ensure that changes are the result of moderate intensity exercise versus other factors such as socialization. In addition, this approach helps to maintain participant retention in the control group.

#### 2.3.3. Exercise Dose and Adherence

Exercise dose was calculated by multiplying the duration (minutes), and frequency (number of days) of each activity performed for all sessions for both groups using previously published metabolic equivalents (METs) in the Compendium of Physical Activities [22]. Exercise dose was expressed as MET∙min∙week^−1^ and averaged over the duration of the intervention. 

Exercise adherence refers to the proportion of exercise or stretching and breathing sessions attended while achieving the prescribed exercise dose; this was tracked electronically via REDCap [23]. Adherence was calculated by dividing the number of sessions attended by the total number of possible sessions within a participant’s gestational period. Participants were considered “exercise adherent” if their attendance was ≥80%. 

### 2.4. Maternal Blood Sample Collection and Analysis

Maternal blood samples were drawn via venipuncture at 16 and 36 weeks of gestation to measure maternal DHA and EPA. The blood samples were collected in anticoagulant tubes and centrifuged at 1000× *g* to separate plasma and red blood cells (RBCs). All blood draws occurred between 6:00 and 8:00 a.m., controlling for the effect of circadian rhythm and fasting overnight (>9 h).

DHA and EPA were extracted from RBCs using Solid Phase Extraction and analyzed using Liquid Chromatography Mass Spectrometry (LC/MS/MS) using previously published methods [24]. The level of PUFAs on red blood cells (RBCs) is a direct measure that provides information based on long-term intake of PUFAs versus plasma levels [25]. Since we did not collect information regarding maternal supplement use (i.e., fish oils or anti-inflammatory substances), we measured DHA and EPA levels in RBCs.

### 2.5. Infant Body Composition Measures

Participants were scheduled for a clinic visit 1 month after the birth of their child. At 1 month of age, neonatal weight (kg), height (cm), body mass index (BMI), abdominal, mid-upper arm and head circumferences (cm), femur, leg, and humeral lengths (cm), and lean mass volume (cm^3^), and BF% were measured in our pediatric clinic. Weight and length (or infant height) were measured using a standard, calibrated infant scale and horizontal stadiometer, respectively. For the skinfold measures, standard skinfold calipers (calibrated Lange calipers) were used to obtain infant BF% as a measurement of body composition. This was assessed using a published protocol and calculation for three-site infant skinfold measures at the triceps, biceps, and subscapular sites. The sum of skinfold thickness data was then used to calculate percent BF using the Slaughter et al. (1988) equation [26]: BF = (1.21·([triceps] + [subscapular])) − ((0.008)·([triceps] + [subscapular])·([triceps] + [subscapular])) − 1.7. Lean mass volume was calculated using the two equations [27]: 1st: Lean Mass = ([weight^0.6469^])·([height^0.7236^]); 2nd: Lean mass volume (cm^3^) = (21.5·[lean mass])·3.8. A standard body tape was used to obtain infant lengths (humeral, femur, leg, and body) and circumferences (head, abdominal, and mid-upper arm). All measures were taken twice on the right side of the infant. All measures were completed at one of our clinic locations by trained exercise physiology students.

### 2.6. Maternal Descriptors and Covariates

Maternal age, parity, gravida, pre-pregnancy BMI, gestational weight gain (GWG), and gestational age were abstracted from various sources including pre-screening eligibility questionnaires and electronic health records. Gestational weight gain was calculated using the standard expression: GWG (kg) = (weight at delivery − weight before pregnancy). In cases of missing weight at delivery, the last recorded study weight (36 weeks of gestation) was used. Pre-pregnancy BMI was calculated using self-reported height (m), and weight (kg) via the following established equation [28]: BMI = ((weight (kg)) ÷ ([height (m^2^)])).

### 2.7. Statistical Analysis

Between-group mean differences for maternal descriptive characteristics, 16- and 36-week measurements of DHA and EPA, and 1-month infant body composition measures of height, weight, circumferences, BF%, and lean body mass were assessed using two-tailed student independent t-tests or Wilcoxon Rank Sum tests, depending on the conditional distribution of the data. Gravida and Parity were not normally distributed and expressed as median (Minimum, Maximum) while all other variables were normally distributed and expressed as Mean ± SD. Additionally, changes in DHA and EPA were calculated via the differences in values measured at 16 and 36 weeks of gestation. Intention-to-treat (ITT) and per protocol analyses were performed. ITT included all participants with complete data. Per protocol included participants that were “exercise adherent” to >80% of exercise sessions and dose with one control participant excluded because she did not follow her group assignment. Spearman correlations were performed to assess associations between maternal exercise dose (MET∙min∙week^−1^), maternal DHA, EPA at 36 weeks of gestation and infant body composition measures at 1 month postnatal. To determine the effect of maternal exercise and DHA and EPA concentrations, as well as infant body composition measures, multiple linear regression analyses were performed. The primary outcome variables were height, weight, lean mass, circumferences, skinfold measures, and BF% at 1 month postnatal. The primary independent variables were maternal exercise dose, as well as DHA and EPA concentrations. The effects of maternal exercise and DHA or EPA were assessed via interaction terms in the regression models. Maternal characteristics assessed as potential covariates in the models included maternal age, pre-pregnancy BMI, gravida, parity, VO_2peak_, GA, GWG, and infant birth weight. The analysis for each primary outcome followed the same structured series of model building: Model 1 = unadjusted main effects model, Model 2 = Model 1 + interaction (inclusion of exercise OR PUFA levels), and Model 3 (depending on the influence of the interaction) = Model 1 or 2 + maternal covariates. Each maternal covariate was entered into the model separately to evaluate its influence on the parameter estimates. Baseline values of each outcome variable were adjusted for their respective regression models. Statistical analyses were performed using SPSS, version 25 (IBM, Cary, NC, USA). Two-tailed statistical significance was set a priori at <0.05.

#### Statistical Power Analysis

For between-group comparisons, a post hoc power analysis revealed we need 18 (9 per group) for the intention-to-treat analysis and 16 (8 per group) for the per protocol analysis with 80% power and 0.05 alpha level to detect differences in triceps skinfold thickness [29]. For our regression analysis, with our total sample of 35 participants, we met the minimum standard of N ≥ 25 in previously published research, as well as 30 total samples to suffice the minimum 5:1 rule for each measurement [30,31].

## 3. Results

### 3.1. Study Population

On average, participants were 31 years old, had a pre-pregnancy BMI of 26.2 kg/m^2^ (overweight), and gained 10.8 kg through pregnancy. All participants delivered full term (37–41 weeks) infants. In the ITT analysis, there were no significant between group differences for maternal age, gravida, parity, pre-pregnancy BMI, pre-intervention VO_2peak_, GWG, and GA. In the ITT analysis, there was not an observed statistical difference of birthweight for infants between groups. In the per protocol analysis, the exercise group had a higher pre-intervention VO_2peak_ (25.6 ± 5.4 vs. 21.6 ± 3.3, *p* = 0.038) and longer gestation compared with controls (40.0 ± 1.1 vs. 39.2 ± 0.7, *p* = 0.048) (Table 1). No other between-group differences were observed. In the per protocol analysis, there was not an observed statistical difference in birthweight for infants between groups. 

### 3.2. Maternal DHA and EPA Concentrations

No between-group differences were observed in maternal concentrations of DHA and EPA at 16 or 36 weeks, or the difference across pregnancy (Table 2); however, at 36 weeks of gestation, exercisers had 65.2% higher levels of DHA and 1.0% lower levels of EPA relative to controls. 

### 3.3. Infant Body Composition Measures 

In the ITT analysis, 1-month-old infants born to women in the aerobic group (n = 25), relative to controls (n = 10), exhibited lower skinfold measurements of the triceps (7.3 ± 1.6 vs. 8.9 ± 1.2, *p* = 0.01), sum of skinfolds (19.8 ± 3.6 vs. 22.8 ± 3.3, *p* = 0.03), and lower body fat percentage (13.7 ± 2.9 vs. 16.1 ± 2.2, *p* = 0.02) (Table 3). In per protocol analysis, 1-month-old infants born to women in the aerobic group (n = 19), relative to controls (n = 10), exhibited lower skinfold measurements of the triceps (7.2 ± 1.6 vs. 8.9 ± 1.2, *p* = 0.008), skinfold measurements of the subscapular (6.3 ± 1.8 vs. 7.8 ± 1.7, *p* = 0.04), sum of skinfolds (19.1 ± 3.5 vs. 22.8 ± 3.3, *p* = 0.01), and lower body fat percentage (13.1 ± 2.7 vs. 16.1 ± 2.2, *p* = 0.006). There were no differences between groups in infant morphometric measures (Table 3). In per protocol analysis, stratification of pre-pregnancy BMI (normal weight < 25.0 and overweight/obese > 25.0), exhibited a significant difference for infants born to normal weight mothers in the aerobic group (n = 11), relative to controls (n = 4), in BF% (12.2 ± 2.3 vs. 15.1 ± 0.5, *p* = 0.03); while no significant difference was seen in the overweight stratification between infants born to mothers in the aerobic (n = 8), relative to controls (n = 6) (14.4 ± 2.9 vs. 16.8 ± 2.7, *p* = 0.14).

### 3.4. Correlations and Regression Analysis

None of the potential covariates (maternal age, pre-pregnancy BMI, gravida, parity, VO_2peak_, GA, GWG, or infant birth weight) impacted the regression coefficients, thus, they were excluded from the models. In an ITT analysis, there was a significant negative correlation between MET∙min∙week^−1^ and infant triceps skinfold thickness (rho_S_ = −0.529; *p* = 0.001), subscapular skinfold thickness (rho_S_ = −0.493; *p* = 0.003), sum of skinfolds (rho_S_ = −0.525; *p* = 0.001), and body fat percentage (rho_S_ = −0.602; *p* ≤ 0.001); no other significant correlations were found between MET∙min∙week^−1^ and 1-month infant measures. There were no significant correlations with measures of differences of DHA or EPA at 1 month postnatal. After controlling for 36-week DHA and EPA levels, we found that increased exercise dose predicted decreased infant triceps skinfold thickness (F (1,33) = 12.378; *p* = 0.001, β = −0.522, 95% CI = −0.005, −0.001), with an r^2^ of 0.27, subscapular skinfold thickness (F (1,33) = 7.937; *p* = 0.008, β = −0.440, 95% CI = −0.005, −0.001), with an r^2^ of 0.19, sum of skinfolds (F (1,33) = 14.133; *p* = 0.001, β = −0.548, 95% CI = −0.012, −0.004), with an r^2^ of 0.28, mid-upper arm circumference (F (1,33) = 4.178; *p* = 0.049, β = −0.335, 95% CI = −0.003, 0.000), with an r^2^ of 0.11, and body fat percentage (F (1,33) = 15.305; *p* = 0.000, β = −0.563, 95% CI = −0.009, −0.003), and with an r^2^ of 0.32 (Table 4). There were no other significant associations with MET∙min∙week^−1^ and 1-month infant body composition. Based on linear regression modeling, there were also no statistically significant associations between maternal DHA and EPA concentrations with MET∙min∙week^−1^ for infant measures of height, weight, lean mass, circumferences, skinfold measures, and BF% at 1 month postnatal (Table 4). 

## 4. Discussion

The purpose of this study was to examine the influence of prenatal aerobic exercise and maternal concentrations of DHA and EPA on infant body composition. We hypothesized that maternal aerobic exercise and higher DHA and EPA concentrations result in improved infant body composition (e.g., decreased body fat %) compared with infants born to non-exercising controls with lower DHA and EPA concentrations. The major findings of the current study suggest that (1) infants exposed to aerobic exercise in utero possessed lower skinfold thicknesses and percent body fat compared with infants of non-exercising pregnant women at 1 month of age; (2) maternal DHA and EPA concentrations did not have a detectable effect on infant body composition at 1 month of age; and (3) prenatal aerobic exercise, but not maternal DHA or EPA concentrations, predicts 1-month infant body composition. 

As hypothesized, the current findings suggest that exercise during pregnancy can reduce the accumulation of fat volume in utero, thus lowering infant subscapular skinfold thickness and BF% [9,10,32,33]. For adults, it is generally accepted that a 5% decrease in BF% is clinically relevant, with some studies showing 2.3% changes in BF% leading to an increased hazards ratio of all-cause mortality [34,35]. The present study showed a 3% difference between groups at 1 month (Table 3). Similarly, increased infant skinfold measures at 6 months of age and 1 year of age predict greater fat mass at 6 years of age [36]. Clapp et al. (2002) examined infant weight and BF% at 5 days after birth following a 3 group prenatal exercise intervention differing in exercise dose (high to low, low to high, and moderate to moderate) throughout pregnancy from 8 weeks of gestation until delivery. They observed that the moderate exercise dose throughout pregnancy also resulted in lower body fat % in infants exposed to exercise in utero [10]. The present RCT completed the exercise intervention in the 2nd and 3rd trimesters of the pregnancy and measured infant body composition at 1 month of age to determine if differences persisted 4 weeks after delivery, observing similar results as Clapp et al. [10]. McDonald et al. examined infant body composition using similar methodology as the present study and observed similar results for infant triceps skinfold thickness and BF% at 1 month of age [37]. Since previous research demonstrated fat mass in infancy to predict greater fat mass up to 6 years of age [36], our results support the benefits of maternal exercise on decreasing infant BF% and potentially the propensity for obesity later in life; thus, these findings add to the literature the importance of aerobic exercise at recommended levels throughout pregnancy on infant BF% after birth. Infant body composition measures can be used to assess the risk of developing obesity later in life [38]. Since skinfold measures in infancy predict fat mass up to 6 years of age, the lower BF% observed among infants exposed to aerobic exercise in utero may provide an effective strategy for decreasing the likelihood of childhood obesity [36].

The current study found that maternal DHA and EPA during pregnancy did not exert a detectable effect on infant body composition. The null effects of maternal DHA and EPA concentrations on infant body composition were unexpected and were inconsistent with the current literature. Previous research suggests that DHA and EPA accumulation in the fetal cells from the maternal diet counteracts the negative effects of omega-6 fatty acids leading to lower infant fat mass, subscapular skinfold thickness, triceps skinfold thickness, and BMI, as well as greater birth weight, lean body mass, and head circumferences; benefits which persist up to 6 years of age [39,40,41,42,43,44,45,46,47,48]. Studies have reported ranges as low as 10 ng/mL to 15,000 ng/mL and all of our subjects fell well-within this range (193 ng/mL to 10,610 ng/mL) [49,50,51,52]. The suggested ratio of omega-6s to omega-3s is 1:1; however, most western diets are 15:1 [53]. DHA and EPA supplementation can help reduce this ratio and lead to the downregulation of pro-inflammatory eicosanoids and reduce adipogenesis. However, if omega-6s, particularly arachidonic acid and linoleic acid, remain high, the effects of DHA and EPA may not be able to overcome the high inflammatory and adipogenic nature of omega-6 fatty acids. While this study employed rigorous measures of maternal DHA and EPA, including measuring DHA and EPA on RBCs, and using fasted samples, our null findings may be attributed to certain nutritional habits of our study population. Firstly, the DHA and EPA concentrations of pregnant participants were likely saturated, potentially the result of women taking prenatal vitamins. The inclusion of DHA and EPA in prenatal vitamins could limit the variability in DHA and EPA RBC concentrations between our study groups. Secondly, we did not assess omega-6s, such as arachidonic acid (ARA), which is considerably higher in western diets and can explain our null findings [48,53]. Interestingly, a previous study found prenatal exercise, not DHA nor EPA, altered maternal triglyceride levels, which is associated with improved birth outcomes [24]

Another unique aspect of this study was the finding that prenatal exercise, not maternal DHA or EPA levels, predicts improved 1-month infant body composition. Our data suggest that lower infant BF% was associated with maternal exercise dose; this finding is further supported by the decrease in infant triceps and subscapular skinfold thicknesses associated with increased maternal exercise dose. Infant humeral circumference was also associated with maternal exercise dose and can directly relate to triceps skinfold thickness. The change in infant skinfold thickness and BF% may be due to maternal epigenetic changes that lead to decreased adipogenesis leading to a decrease in fat cell number and size [54,55]. Interestingly, maternal chronic inflammation is associated with fetal mesenchymal cell differentiation towards adipogenesis (fat cell development) [56], and infant BF% [57]; whereas, exercise is considered anti-inflammatory, and thus may explain the decreased adipose volume. Previous research found low maternal physical activity positively correlated to infant birth weight and fat mass in women with high maternal BMI [2,3]. Although prenatal exercise dose exhibits a negative effect on infant triceps and subscapular skinfold thickness, sum of skinfolds, humeral circumference, and infant body fat %, those relationships were not influenced by DHA or EPA. These findings are similar to a previous study which found prenatal exercise dose, not DHA nor EPA, predicted maternal triglyceride levels [24]. Possible explanations for these null findings are the null associations between DHA and EPA on infant body composition and the small sample size. Since this study did not find a relationship between DHA and EPA on infant body composition, the possibility of these measures influencing regression analyses was minimized. Moreover, our small sample reduced the statistical power to observe a significant effect. Overall, the finding that exercise dose and not DHA nor EPA predict improved 1-month infant body composition is encouraging in light of the increasing trend of childhood obesity; thus, exercise during pregnancy can offer a low-cost, non-pharmacological, easy-to-implement intervention that can improve the future health of the next generation. 

This study has strengths that warrant mention. First, the study employed a randomized controlled design, providing the strongest evidence for causality. Second, we utilized a supervised exercise intervention meeting the recommended level of exercise during pregnancy. Third, our quantification of DHA and EPA utilized the sensitive and precise technique of targeted metabolomics using SPE and LC/MS/MS. Fourth, the measurement of DHA and EPA from RBCs provides a long-term picture of PUFAs in the maternal system. In addition to strengths, there were several potential limitations. Firstly, many samples were excluded or missing primarily due to non-collection or non-fasted samples, consequently yielding a small analytical sample and reducing statistical power. Second, our observed sample population included healthy participants with uncomplicated pregnancies, thus decreasing the generalizability of the findings; however, the excessive GWG does mirror what is often seen in pregnant women in the US. Third, we did not assess omega-6s which may have counteracted omega-3 effects, since most western diets have a 15:1 omega-6s/omega-3s. Fourth, although breastfed infants tend to be leaner than formula fed infants, we did not assess the influence of breastfeeding on offspring outcomes; however, all infants were breastfed at this point, which minimizes this variable [58]. Lastly, we did not measure any epigenetic factors and thus were unable to compare and contrast their possible effects on the results. There can be a potential difference in response between weight-bearing treadmill walking vs. non-weight-bearing cycling; however, this difference was minimized by maintaining the same exercise dose throughout pregnancy. Lastly, potential differences between measured concentrations compared with other studies are likely due to the differences in measuring techniques as many studies utilize liquid–liquid extractions with gas chromatography compared with our use of SPE and LC/MS/MS.

## 5. Conclusions

The current study strengthens the existing evidence with respect to the positive effects of prenatal exercise on 1-month infant body composition. Importantly, this observation suggests prenatal exercise may effectively decrease measures of body fat in infants. Unexpectedly, our study showed no statistically significant association between maternal DHA/EPA levels on infant morphometric measures, a finding contrary to the existing scientific literature. Moreover, our study observed that exercise dose, not maternal DHA nor EPA levels, predict improved 1-month infant body composition. Future studies should consider assess the effects of different exercise modalities (treadmill vs. cycling) as well as exercise dose in order to help reduce infant fat accumulation, thus preventing negative infant outcomes associated with long-term and chronic diseases such as obesity.

## Figures and Tables

**Table 1:** Intention-to-treat and per protocol analysis of maternal demographics between aerobic exercise and non-exercising controls.

	Intention-to-Treat			Per Protocol		
	Control (n = 10)	Exercise (n = 25)	*p*-Values	Control (n = 10)	Exercise (n = 19)	*p*-Values
Maternal Measures						
Maternal Age (years)	30.5 ± 4.5	32.0 ± 4.5	0.37	30.5 ± 4.5	31.3 ± 4.0	0.62
Gravida (# of pregnancies) ^a^	2 (1, 4)	2.5 (1, 4)	0.26	2 (1, 4)	2 (1, 4)	0.20
Parity (# of births) ^a^	1 (0, 2)	0.5 (0, 2)	0.22	1 (0, 2)	1 (0, 2)	0.15
Pre-Pregnancy BMI (kg/m^2^)	28.2 ± 5.0	25.5 ± 4.4	0.13	28.2 ± 5.0	25.5 ± 4.6	0.15
Pre-Intervention VO_2peak_ (mL∙kg^−1^∙min^−1^)	21.6 ± 3.3	24.2 ± 5.7	0.17	21.6 ± 3.3	25.6 ± 5.4	0.04 *
GWG (kg)	10.9 ± 4.2	14.0 ± 4.4	0.06	10.9 ± 4.2	14.1 ± 4.4	0.08
GA (weeks)	39.2 ± 0.7	40.0 ± 1.7	0.20	39.2 ± 0.7	40.0 ± 1.1	0.048 *
Infant Measures						
Infant Sex (%male)	75.0%	50.0%		75.0%	46.2%	
Birth weight (kg)	3.4 ± 0.4	3.7 ± 0.1	0.20	3.4 ± 0.4	3.7 ± 0.3	0.11

Values with normal distribution expressed as Mean ± SD; ^a^ not normally distributed, expressed as median (Minimum, Maximum); #: Number, BMI: Body Mass Index; VO_2peak_: Peak Volume of Oxygen Consumption; GWG: Gestational Weight Gain; GA: Gestational Age at birth * *p* < 0.05.

**Table 2:** Intention-to-treat and per protocol analysis of maternal DHA and EPA red blood cell concentrations between aerobic exercise and non-exercising controls.

	Intention-to-Treat			Per Protocol		
	Control (n = 10)	Exercise (n = 25)	*p*-Values	Control (n = 10)	Exercise (n = 19)	*p*-Values
16 Week						
DHA (ng/mL)	1926 ± 1120.2	2572.5 ± 2576.5	0.45	1926 ± 1120.2	2493.3 ± 2786.8	0.55
EPA (ng/mL)	957.9 ± 252.6	1073.9 ± 394.5	0.36	957.9 ± 252.6	1044.0 ± 348.7	0.27
36 Week						
DHA (ng/mL)	2019.1 ± 1676.3	2815.7 ± 2492.0	0.40	2019.1 ± 1676.3	3096.5 ± 2782.3	0.50
EPA (ng/mL)	1424.4 ± 1201.2	1269.4 ± 1259.4	0.74	1424.4 ± 1201.2	1405.7 ± 1422.6	0.97
Difference						
DHA (ng/mL)	93.0 ± 2188.2	243.2 ± 3030.2	0.88	93.0 ± 2188.2	603.2 ± 3341.2	0.67
EPA (ng/mL)	466.6 ± 1283.1	195.5 ± 1239.4	0.57	466.6 ± 1283.1	361.6 ± 1374.2	0.84

Values with normal distribution expressed as Mean ± SD; DHA: Docosahexaenoic Acid; EPA: Eicosapentaenoic Acid.

**Table 3:** Intention-to-treat and per protocol analyses of infant body composition measures between aerobic exercise and non-exercising controls.

	Intention-to-Treat			Per Protocol		
	Control (n = 10)	Aerobic (n = 25)	*p*-Value	Control (n = 10)	Aerobic (n = 19)	*p*-Value
Height (cm)	53.9 ± 2.7	54.4 ± 3.7	0.73	53.9 ± 2.7	54.3 ± 2.9	0.73
Weight (kg)	4.6 ± 0.6	4.5 ± 0.5	0.66	4.6 ± 0.6	4.5 ± 0.5	0.43
BMI	15.9 ± 1.5	15.5 ± 2.0	0.53	15.9 ± 1.5	15.2 ± 1.9	0.31
Skinfolds						
Triceps (mm)	8.9 ± 1.2	7.3 ± 1.6	0.01 *	8.9 ± 1.2	7.2 ± 1.6	0.008 **
Subscapular (mm)	7.8 ± 1.7	6.8 ± 1.9	0.17	7.8 ± 1.7	6.3 ± 1.8	0.04 *
Bicep (mm)	6.2 ± 1.7	5.7 ± 1.6	0.48	6.2 ± 1.7	5.6 ± 1.7	0.43
Sum of Skinfolds (mm)	22.8 ± 3.3	19.8 ± 3.6	0.03 *	22.8 ± 3.3	19.1 ± 3.5	0.01 *
Body Fat %	16.1 ± 2.2	13.7 ± 2.9	0.02 *	16.1 ± 2.2	13.1 ± 2.7	0.006 **
Lean Body Volume (cm^3^)	3948.3 ± 443.4	3922.6 ± 416.5	0.87	3948.3 ± 443.4	3873.6 ± 365.9	0.63
Circumferences						
Abdominal (cm)	39.0 ± 2.3	38.7 ± 2.8	0.76	39.0 ± 2.3	38.4 ± 3.0	0.56
Head (cm)	37.1 ± 1.4	37.7 ± 2.6	0.36	37.1 ± 1.4	37.5 ± 2.1	0.53
Mid-Upper Arm (cm)	11.7 ± 1.4	11.3 ± 1.0	0.32	11.7 ± 1.4	11.3 ± 1.1	0.38
Head/Abdominal	0.95 ± 0.03	0.98 ± 0.1	0.32	0.95 ± 0.03	0.98 ± 0.1	0.29
Lengths						
Femur (cm)	10.3 ± 1.1	11.0 ± 1.6	0.21	10.3 ± 1.1	11.2 ± 1.7	0.17
Leg (cm)	21.9 ± 1.9	22.5 ± 1.9	0.37	21.9 ± 1.9	22.4 ± 2.1	0.46
Humeral (cm)	8.8 ± 0.7	9.0 ± 1.2	0.60	8.8 ± 0.7	9.2 ± 1.3	0.40

Values with normal distribution expressed as mean ± SD; BMI: Body Mass Index; * *p* < 0.05; ** *p* < 0.01.

**Table 4:** Multiple linear regression models of maternal exercise dose, DHA, and EPA concentrations influence on infant body composition measures at 1 month of age.

		β (95% CI)	*p*-Value
MET∙min∙week^−1^	Height (cm)	0.015 (−0.004, 0.005)	0.93
	Weight (kg)	−0.229 (−0.001, 0.000)	0.19
	BMI (kg/m^2^)	−0.240 (−0.004, 0.001)	0.16
	Triceps Skinfold (mm)	−0.522 (−0.005, −0.001)	0.001 **
	Subscapular Skinfold (mm)	−0.440 (−0.005, 0.001)	0.008 **
	Biceps Skinfold (mm)	−0.230 (−0.003, 0.001)	0.18
	Sum of Skinfolds (mm)	−0.548 (−0.012, −0.004)	0.001 **
	Body Fat %	−0.563 (−0.009, −0.003)	0.000 ***
	Lean Body Mass (volume-cm^3^)	−0.157 (−0.791, 0.300)	0.37
	Abdominal Circumference (cm)	−0.165 (−0.005, 0.002)	0.34
	Head Circumference (cm)	0.166 (−0.001, 0.003)	0.34
	Arm Circumference (cm)	−0.335 (−0.003, 0.000)	0.049 *
	Femur Length (cm)	0.333 (0.000, 0.004)	0.051
	Leg Length (cm)	0.018 (−0.002, 0.003)	0.92
	Humeral Length (cm)	−0.031 (−0.002, 0.001)	0.86
	Head/Abdominal Circumference	0.295 (0.000, 0.000)	0.09
36 Week DHA Concentration	Height (cm)	−0.003 (−0.001, 0.001)	0.99
	Weight (kg)	0.017 (0.000, 0.000)	0.93
	BMI (kg/m^2^)	0.029 (0.000, 0.000)	0.87
	Triceps Skinfold (mm)	−0.048 (0.000, 0.000)	0.78
	Subscapular Skinfold (mm)	−0.036 (0.000, 0.000)	0.84
	Biceps Skinfold (mm)	0.054 (0.000, 0.000)	0.76
	Sum of Skinfolds (mm)	−0.016 (−0.001, 0.001)	0.93
	Body Fat %	−0.062 (−0.001, 0.000)	0.73
	Lean Body Mass (volume-cm^3^)	0.006 (−0.063, 0.066)	0.97
	Abdominal Circumference (cm)	−0.227 (−0.001, 0.000)	0.19
	Head Circumference (cm)	−0.222 (0.000, 0.000)	0.20
	Arm Circumference (cm)	−0.128 (0.000, 0.000)	0.46
	Femur Length (cm)	0.074 (0.000, 0.000)	0.67
	Leg Length (cm)	0.159 (0.000, 0.000)	0.36
	Humeral Length (cm)	0.256 (0.000, 0.000)	0.14
	Head/Abdominal Circumference	0.061 (0.000, 0.000)	0.73
36 Week EPA Concentration	Height (cm)	0.006 (−0.001, 0.001)	0.97
	Weight (kg)	−0.160 (0.000, 0.000)	0.36
	BMI (kg/m^2^)	−0.155 (−0.001, 0.000)	0.38
	Triceps Skinfold (mm)	−0.210 (−0.001, 0.000)	0.23
	Subscapular Skinfold (mm)	−0.236 (−0.001, 0.000)	0.17
	Biceps Skinfold (mm)	−0.189 (−0.001, 0.000)	0.28
	Sum of Skinfolds (mm)	−0.291 (−0.002, 0.000)	0.09
	Body Fat %	−0.273 (−0.001, 0.000)	0.11
	Lean Body Mass (volume-cm^3^)	−0.112 (−0.158, 0.082)	0.52
	Abdominal Circumference (cm)	−0.137 (−0.001, 0.000)	0.43
	Head Circumference (cm)	0.124 (0.000, 0.001)	0.48
	Arm Circumference (cm)	−0.147 (0.000, 0.000)	0.40
	Femur Length (cm)	−0.043 (0.000, 0.000)	0.81
	Leg Length (cm)	−0.104 (−0.001, 0.000)	0.55
	Humeral Length (cm)	−0.125 (0.000, 0.000)	0.48
	Head/Abdominal Circumference	0.208 (0.000, 0.000)	0.23

MET: metabolic equivalents of exercise; DHA: Docosahexaenoic Acid; EPA: Eicosapentaenoic acid; BMI: Body Mass Index; * *p*-value < 0.05; ** *p*-value < 0.01; *** *p*-value < 0.001.

## Data Availability

Not applicable.

## Abbreviations

ACN	Acetonitrile
ACOG	American College of Obstetrics and Gynecology
ACSM	American College of Sports Medicine
AHA	American Heart Association
BF%	Body Fat Percentage
CDC	Center for Disease Control
DHA	Docosahexaenoic Acid
EPA	Eicosapentaenoic Acid
GA	Gestational Age
GWG	Gestational Weight Gain
H_2_O	Water
HPLC	High Performance Liquid Chromatography
HR	Heart Rate
IPA	Isopropanol
LC/MS/MS	Liquid Chromatography Tandem Mass Spectrometry
LOD	Limit of Detection
LOQ	Limit of Quantification
MeOH	Methanol
MET	Metabolic Equivalent
PT	Physical Therapy
PUFA	Polyunsaturated Fatty Acid
RBC	Red Blood Cell
RPE	Rating of Perceived Exertion
S/N	Signal-to-Noise ratio
SPE	Solid Phase Extraction
THR	Target Heart Rate
VO_2peak_	Peak Volume of Oxygen Consumption
